# Eyelid Molluscum Contagiosum Lesions in Two Patients with Unilateral Chronic Conjunctivitis

**DOI:** 10.4274/tjo.52138

**Published:** 2017-08-15

**Authors:** Şule Serin, Ayşe Bozkurt Oflaz, Pınar Karabağlı, Şansal Gedik, Banu Bozkurt

**Affiliations:** 1 Selçuk University Faculty of Medicine, Department of Ophthalmology, Konya, Turkey; 2 Selçuk University Faculty of Medicine, Department of Pathology, Konya, Turkey

**Keywords:** Molluscum contagiosum, eyelid lesions, chronic conjunctivitis

## Abstract

Molluscum contagiosum (MC) is a viral infection of the skin and mucosal tissues characterized by skin-colored or transparent round nodules with a dimple or pit in the center. The infection is caused by a DNA poxvirus called the MC virus. Although MC generally occurs in children, it has also been reported in immunocompromised and atopic patients. The virus is transmitted by skin contact or sexual intercourse. The lesions disappear spontaneously within several months in most cases. However, excision, cryotherapy, cauterization, topical chemical and antiviral agents, and/or oral cimetidine are used in refractory cases or to accelerate the healing process. Herein, we discussed the clinical findings and our treatment of two patients with unilateral chronic conjunctivitis associated with eyelid MC lesions in light of the literature.

## INTRODUCTION

Molluscum contagiosum (MC) is characterized by papular lesions in the skin and mucous membranes caused by the *Molluscum contagiosum* virus, a DNA virus from the poxvirus group.^1,2^ Humans are the only known host. The virus is transmitted through skin contact and sexual intercourse. It is particularly common in hot, developing countries and in communities with poor personal hygiene.^1,2,3,4^ Signs of MC infection have been reported in about 4.5% of children under 10 years old. In developed countries, infections are sometimes acquired from swimming pools, saunas, and sports centers. In addition, it can also be seen in immunosuppressed patients (e.g. with AIDS or using drugs such as corticosteroids, TNF-α antibodies, and methotrexate), and in patients with atopic dermatitis, sarcoidosis, and Wiskott-Aldrich syndrome.^5,6,7,8^ Diagnosis is usually based on clinical findings, and histopathological examination and laboratory tests are often not necessary.

The lesions, located on the skin and mucosa, typically appear as small (between about 2-6 mm), raised, flesh-colored or clear papules and there is pearly white caseous material in the pitted center.^1,2,3,9^ The papular lesions, usually found in clusters, are often seen on the face, head, torso, and extremities in children, and in the genital area, lower abdomen, and upper legs in young adults in whom the infection is sexually transmitted.

Ophthalmic MC lesions are often located on the eyelids.^1,2,8,9,10^ Viral proteins shed from the lid lesions into the tear film can lead to a hypersensitivity reaction with secondary chronic follicular reaction, punctate keratopathy in the conjunctiva, and in some cases subepithelial opacities and pannus. Rarely, primary MC lesions are seen in the conjunctiva and cornea. While the lesions usually resolve spontaneously within a few months, treatments such as excision, incision and curettage, cryotherapy, cauterization, topical chemical agents, and oral cimetidine can be used in refractory cases and to speed up the healing process.^1,11,12,13,14^

In this case report, the clinical findings and treatment of two patients who were admitted with unilateral chronic conjunctivitis and were found to have eyelid MC lesions are discussed in light of the literature.

## CASE REPORTS

### Case 1

A 5-year-old female patient who had raised lesions on her right eyelid for about 3 months and complaints of redness and watering in her right eye was admitted to our clinic. It was learned that she had previously been seen by three different ophthalmologists and had been treated with topical ofloxacin (Exocin® 4 times daily), olopatadine HCl (Patanol® 2 times daily), and dexamethasone sodium phosphate (Dexa-sine® 3 times daily) for 2 months. Visual acuity measured with E chart was 20/20 in both eyes. Intraocular pressure (IOP) was 12 mmHg in both eyes. On anterior segment examination, 2 pitted papular lesions were detected on the right upper eyelid 2 mm from the lash line and a similar lesion was noted 5 mm from the lash line on the lateral aspect of the lid (Figure 1A). The conjunctiva was hyperemic and there was intense follicular reaction in the lower fornix (Figure 1B). Suspecting MC, the existing treatment was discontinued and topical ganciclovir (Virgan® gel 3 times daily), lubricant treatment (Tears Naturale Free® drops 5 times daily), and eyelash cleansing were recommended. Complete blood count and immunoglobulin levels were normal. The family was informed that the lesions may spontaneously regress, and would be surgically excised if they did not. We observed in follow-up examination 1 month later that the lesions and symptoms had not regressed, so the papular lesions were excised preserving the integrity of the cyst wall and cryotherapy was applied to the base of the lesions. The detection of inclusion bodies in the material sent to pathology confirmed a diagnosis of MC (Figure 1C, 1D). The patient was treated postoperatively with topical moxifloxacin (Vigamox®) drops 5 times daily for 2 weeks and lubricant eye drops (Tears Naturale Free® 5 times daily). At 1-month follow-up, the lid lesions had disappeared and the follicular reaction was reduced. The patient remains under follow-up and no recurrence has been seen for 6 months (Figure 1E, 1F).

### Case 2

A 24-year-old female patient presented to our clinic with complaints of swelling of the right upper eyelid and redness in the eye for 2 months. Her visual acuity was 20/20 in both eyes and IOP was 14 mmHg. On anterior segment examination, there was a pitted papular lesion 2x2 mm in size on the medial aspect of the right upper eyelid at a distance of 4 mm from the lash line, and another small papule just below the eyebrow (Figure 2A). There was conjunctival hyperemia in the right eye and a mild to moderate degree of follicular reaction in the conjunctiva (Figure 2B). Serological tests for HIV and hepatitis A, B, and C viruses were negative. Immunoglobulin levels and lymphocyte subtype values were within normal limits. To reduce the viral load in the patient’s eye, a half-strength dilution of betadine (5%) was instilled and washed out after 30 seconds. Treatment was initiated with topical moxifloxacin (Vigamox® drops 3 times daily), topical ganciclovir (Virgan® gel twice daily), and lubricant therapy (Tears Naturale Free® drops 5 times daily). Follow-up examination 3 weeks later showed that the lesion and ocular symptoms had not regressed, so the larger papule was excised preserving the integrity of the cyst wall and cryotherapy was applied to the base of the lesion. Cryotherapy was applied directly to the smaller papule. Histopathological examination revealed inclusion bodies (Figure 2C). At postoperative 1 month follow-up, the lid lesions had disappeared and the conjunctival follicular reaction was reduced (Figure 2D, 2E). Follow-up is ongoing and no recurrence has been observed for approximately 5 months.

## DISCUSSION

Ocular involvement of MC presents with round, small, hard papules on the eyelids. The virus proliferates in epithelial cells. After the lesions reach about 3-5 mm in diameter, a central depression forms due to the cellular damage mechanism and they typically develop the appearance of whitish lesions filled with caseous material and having a dimple or pit in the center.^1,2^

Although the diagnosis is made clinically when these characteristic MC lesions are evident on the lids, half of patients cannot be diagnosed at the first examination. Both of our patients presented with a 2- to 3-month history of red eye and lid edema. The first case had previously seen several ophthalmologists, but the lid lesions were overlooked and her condition had been treated as infectious conjunctivitis. Charteris et al.^2^ examined the clinical and immunopathologic features of 35 MC cases and found that only 60% of the patients were diagnosed at the time of initial presentation. Histopathologic sections showed increased numbers of T lymphocyte cells and macrophages in the epidermis and dermis around the molluscum lesion.

In recent years, the increased incidence of MC among adults has been associated with AIDS.^1,2,5,6^ HIV-related lesions are usually numerous and at least 5 mm in size. They respond poorly to treatment and recurrence is common. Blood testing of our patients revealed no significant systemic pathology or immunodeficiency.

The differential diagnosis of lid lesions can include basal cell carcinoma, papilloma, chalazion, sebaceous cyst, keratoacanthoma, blepharitis, wart, eczema, obstructed nasolacrimal duct, and ectropion. However, the typical appearance of papular lesions is consistent with MC. Allergic, bacterial, viral, and chlamydial conjunctivitis may be considered in the differential diagnosis of the follicular reaction in patients with conjunctival involvement. For patients with corneal pannus, it may be necessary to rule out chlamydia and rosacea keratitis in the differential diagnosis.

Chlamydial conjunctivitis is defined as inclusion conjunctivitis occurring in adulthood. Ocular involvement may occur in 0.3-2% of patients with urogenital manifestation.^15^ Ocular findings emerge within 5-14 days after contagion. Follicular response appears in the lower fornix and palpebral conjunctiva. In chlamydial infection, history and clinical findings facilitate diagnosis, and treatment is systemic.^15^

Although MC resolves spontaneously within months in healthy, immunocompetent individuals, it requires treatment in refractory cases.^1^ It should be kept in mind that progressive corneal neovascularization and scarring may develop, especially in patients with chronic conjunctivitis and corneal involvement. Conjunctivitis and keratitis show rapid improvement after skin lesions are eradicated. There is no known antiviral treatment specific for MC. While topical ganciclovir is approved for use in acute herpetic keratitis, its efficacy has also been demonstrated against cytomegalovirus and some strains of adenovirus, while systemic administration of ganciclovir is effective against HIV.^16^ Although there are no previous studies showing that topical ganciclovir is effective against MC, we initially administered it to both of our patients considering its broad-spectrum antiviral activity, but no improvement was achieved.

Surgical techniques include cryotherapy with liquid nitrogen, cauterization, needle aspiration, photodynamic therapy, and various laser therapies.^1,11,12,13,14^ Silver nitrate, phenol, and trichloracetic acid are used as chemical agents, while treatment options for immunocompromised patients with refractory lesions include topical cidofovir (5%), intralesional or systemic interferon, imiquimod cream (5%), salicylic acid, glycolic acid, tretinoin, tazarotene, 5% sodium nitrate, podophyllotoxin, liquefied phenol, cantharidin, potassium hydroxide, 1% adenine cream, and oral cimetidine. Eyelid scarring, depigmentation, and eyelash loss may occur after these treatments. Karabulut et al.^11^ reported dramatic improvement in dense eyelid lesions with only physical drainage in an immunocompetent pediatric patient with MC. Weller et al.^14^ compared esthetic appearance after chemical ablation with phenol versus emptying the lesion by squeezing and reported that although there was no significant difference, phenol treatment caused more scarring. In our cases, we demonstrate that surgical excision of the papules and cryotherapy applied to their bases and margins resulted in complete disappearance of the lesions with no scarring or recurrences within the 6-month follow-up period, and the chronic conjunctivitis also resolved completely.

## Figures and Tables

**Figure 1A:**
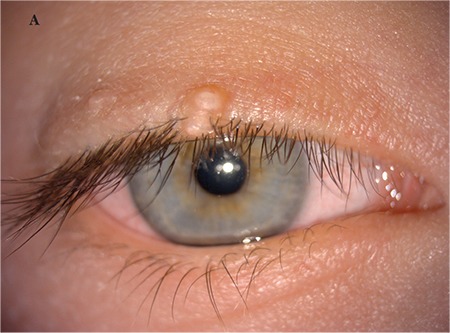
Image of patient 1 showing 2 pitted papular lesions situated 2 mm from the lash line of the right upper lid and a similar lesion 5 mm from the lash line on the lateral aspect of the lid

**Figure 1B:**
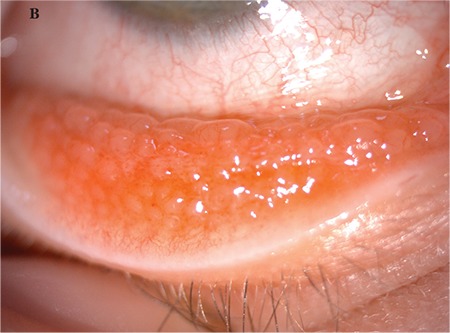
Image of patient 1 showing conjunctival hyperemia and intense follicular reaction at the lower fornix

**Figure 1C, 1D:**
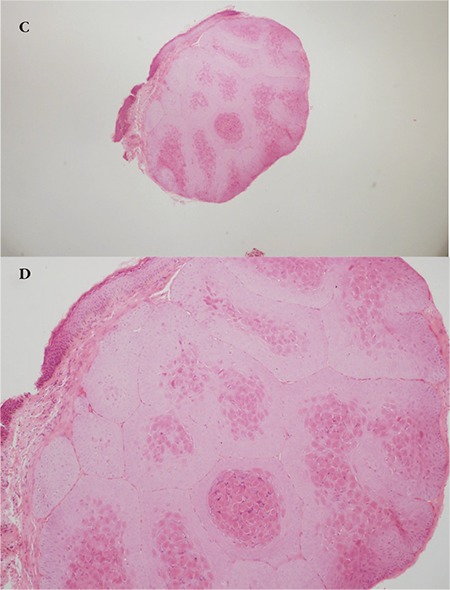
Pathological specimen showing excised papule and eosinophilic intracytoplasmic viral inclusion bodies (hematoxylin & eosin, x20 and x100)

**Figure 1E, 1F:**
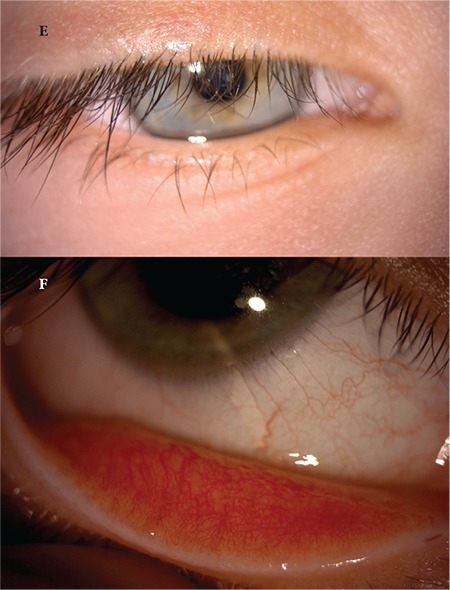
Postoperative images of patient 1 show the eyelid lesion has disappeared and the follicular reaction in the conjunctiva has completely regressed

**Figure 2A:**
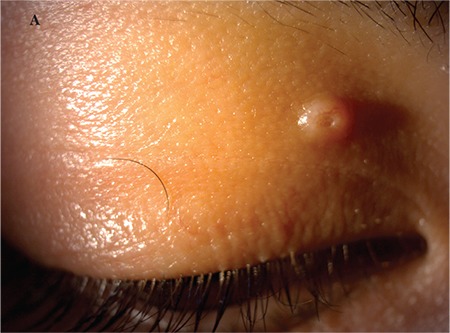
Image of patient 2 showing a pitted papular lesion 2x2 mm in size situated 4 mm from the lash line on the medial aspect of the right upper eyelid

**Figure 2B:**
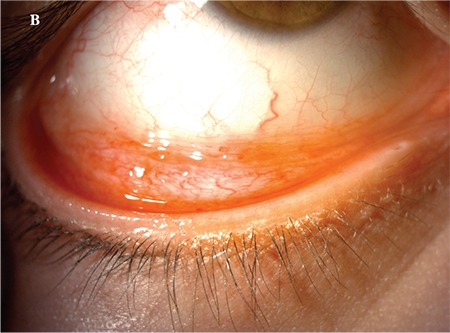
Image of patient 2 showing conjunctival hyperemia and mild to moderate follicular reaction in the tarsal conjunctiva

**Figure 2C:**
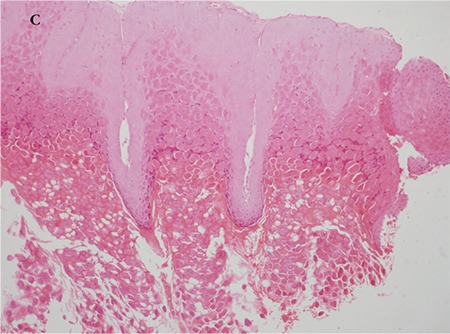
Eosinophilic inclusion bodies are observed in the cytoplasm of squamous cells in the stratum granulosum layer (hematoxylin&eosin, x100)

**Figure 2D, 2E:**
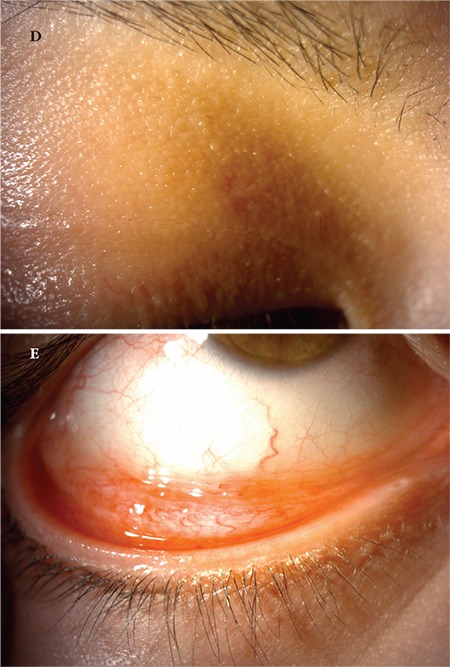
Postoperative images of patient 2 show the lesion on the eyelid has disappeared and the follicular reaction in the conjunctiva is decreased
